# Hepatorenal Syndrome: A Critical Complication in Advanced Cirrhosis

**DOI:** 10.7759/cureus.85488

**Published:** 2025-06-06

**Authors:** Venkata Yashashwini Maram Reddy, Krishna Reddy Maramreddy

**Affiliations:** 1 Internal Medicine, Guntur Medical College, Guntur, IND; 2 Obstetrics and Gynecology, Sai Nursing Home, Darsi, IND

**Keywords:** acute kidney injury, ascites, cirrhosis, hepatorenal syndrome, liver transplantation, portal hypertension, splanchnic vasodilation, vasoconstrictors

## Abstract

Hepatorenal syndrome (HRS) is a life-threatening complication of advanced liver disease, especially cirrhosis. It involves functional kidney failure without structural damage. The main cause is severe splanchnic vasodilation from portal hypertension, which reduces effective blood volume. This triggers compensatory vasoconstriction, leading to reduced kidney perfusion. HRS is classified into two types. Type 1 is rapid and often triggered by infection or bleeding. Type 2 progresses slowly and is linked to refractory ascites. Diagnosis is clinical and requires ruling out shock, nephrotoxins, and kidney disease. Early diagnosis is vital due to its high mortality. We describe the case of a male patient, age 54, with a history of alcohol-induced cirrhosis. He had laboratory evidence of acute kidney injury, oliguria, hyponatremia, and progressive fatigue. Ultrasound showed a nodular liver with ascites, indicating cirrhosis and portal hypertension. Both kidneys retained their normal size and corticomedullary differentiation, ruling out intrinsic renal disease. Type 1 HRS was identified after all other possible causes of renal dysfunction were ruled out. Renal function partially improved after the patient received intravenous albumin and terlipressin. He was then referred to be evaluated for a liver transplant. This case highlights how complex the clinical and diagnostic aspects of HRS can be while also emphasizing the critical need for early detection and treatment. By combining sonographic findings, such as indicators of portal hypertension, with clinical and biochemical data, we can make a more informed diagnosis of HRS. Although pharmacological treatments may provide temporary relief, liver transplantation remains the gold standard for ensuring long-term survival in patients with advanced liver disease complicated by HRS.

## Introduction

Hepatorenal syndrome (HRS) is a severe and life-threatening complication of advanced liver disease, particularly cirrhosis, characterized by functional renal failure without structural kidney damage [1]. According to the most recent International Club of Ascites (ICA) guidelines, HRS is now classified under hepatorenal syndrome-acute kidney injury (HRS-AKI), defined as an increase in serum creatinine ≥0.3 mg/dL within 48 hours or a ≥50% increase from baseline within the past seven days in patients with cirrhosis, ascites, and no other identifiable cause of kidney injury [2]. HRS is classified into two types: type 1, which presents as acute and rapidly progressive kidney failure, and type 2, which progresses more slowly but still carries a poor prognosis [2,3]. AKI occurs in 20-53% of hospitalized patients with decompensated cirrhosis, acute-on-chronic liver failure, or acute liver failure and is associated with increased mortality. Up to 25% of cirrhotic patients who recover go on to develop chronic kidney disease. About 40% of patients with cirrhosis and ascites develop HRS within five years [2]. Common precipitants of HRS include spontaneous bacterial peritonitis, large-volume paracentesis without albumin support, gastrointestinal bleeding, and overuse of diuretics. In liver cirrhosis, progressive portal hypertension and splanchnic arterial vasodilation lead to effective arterial hypovolemia and hyperactivation of vasoconstrictive systems, with dysregulation of the renin-angiotensin system, particularly the balance between the profibrotic ACE-Ang II-AT1 axis and the protective ACE2-Ang-(1-7)-Mas receptor pathway, contributing to circulatory dysfunction, liver fibrosis, and the pathogenesis of HRS [3]. Treatment options for HRS include vasoconstrictors [4] and albumin [4,5], with the combination of terlipressin and albumin restoring kidney function in 40-50% of cases, although overall therapeutic options remain limited [2]. Additional interventions include transjugular intrahepatic portosystemic shunt (TIPS) [6], kidney replacement therapy (KRT) [7], and, ultimately, liver transplantation [8,9]. Early recognition using specific biomarkers [10,11] and timely management are crucial, as HRS is associated with high mortality [12].

## Case presentation

A 54-year-old man of Asian descent presented to the emergency department with reduced urine output for two days and progressive fatigue for two days. The patient denied dysuria, hematuria, or urinary discomfort. His previous medical history includes cirrhosis due to chronic alcoholism, ascites, hypertension, and type 2 diabetes mellitus. He was on furosemide 20 mg, hydrochlorothiazide 20 mg, metformin 500 mg, and glimepiride 1 mg at the time of presentation. He denied any other significant medical conditions and surgeries. He had been consuming three beers daily for 10 years but quit two years ago and was a chronic smoker with a 30-pack-year history. He is a farmer by profession and belongs to a low socioeconomic status. His appetite and sleep are poor; he has normal bowel movements. There is no family history of liver disease or other relevant conditions. 

On the general examination, he is alert and oriented to time, place, and person. He is poorly built and nourished. Clinical examination revealed icterus of the bilateral upper bulbar conjunctiva. There was no evidence of pallor, cyanosis, clubbing, lymphadenopathy, or pedal edema. Vital signs were stable with a pulse rate of 72 bpm, a blood pressure of 100/70 mmHg, and a respiratory rate of 16/min, and the patient was afebrile.

The examination of the oral cavity, including the teeth, gums, tongue, nose, and tonsils, was unremarkable. The abdomen appeared distended with visible flank fullness, and all quadrants moved symmetrically with respiration. The umbilicus was centrally positioned, and the overlying skin appeared stretched and shiny. Dilated veins radiating from the umbilicus were noted, consistent with caput medusae. The external genitalia and hernial orifices were normal. There was no local rise in temperature, tenderness, guarding, rigidity, or rebound tenderness. The liver was palpable 10 cm below the right costal margin in the midclavicular line, was firm and nodular in consistency, and moved with respiration. The spleen was palpable 5 cm below the left costal margin in the midclavicular line, was firm in consistency, and also moved with respiration. Percussion elicited a fluid thrill, suggestive of ascites. Bowel sounds were normal on auscultation, and no bruits were heard. Examination of the cardiovascular system revealed normal S1 and S2 heart sounds with no audible murmurs. Respiratory system assessment showed normal bilateral vesicular breath sounds without any added sounds. Central nervous system examination was within normal limits, with no focal neurological deficits noted.

Liver function tests

Liver function tests revealed significant hepatocellular injury, with elevated aminotransferases: alanine aminotransferase (ALT) at 100 U/L and aspartate aminotransferase (AST) at 250 U/L. Bilirubin levels were markedly elevated, with total bilirubin at 8 mg/dL, direct fraction at 4.1 mg/dL, and indirect fraction at 3.9 mg/dL, consistent with hepatic dysfunction. The patient's synthetic liver function was compromised, as evidenced by a prolonged prothrombin time (PT) of 20 seconds and an international normalized ratio (INR) of 4. Serum albumin was also reduced to 2 g/dL, further supporting impaired hepatic synthetic capacity. These findings are summarized in Table 1 and align with a diagnosis of decompensated cirrhosis in the context of chronic alcohol-related liver disease.

**Table 1:** Liver function test results showing elevated AST, ALT, and GGT levels, indicating hepatocellular injury. Hyperbilirubinemia (both direct and indirect) reflects cholestatic and hepatocellular dysfunction. A prolonged PT and elevated INR suggest impaired hepatic synthetic function. Hypoalbuminemia further supports the diagnosis of decompensated cirrhosis ALT: alanine aminotransferase; AST: aspartate aminotransferase; GGT: gamma-glutamyl transpeptidase; PT: prothrombin time; INR: international normalized ratio

Test	Observed value	Reference range
ALT	100 U/L	10-40 U/L
AST	250 U/L	12-38 U/L
Alkaline phosphatase	110 U/L	25-100 U/L
GGT	80 U/L	8-61 U/L
Total bilirubin	8 mg/dL	0.1-1 mg/dL
Direct bilirubin	4.1 mg/dL	0.0-0.3 mg/dL
Indirect bilirubin	3.9 mg/dL	0.2-0.8 mg/dL
PT	20 sec	11-15 sec
INR	4	0.8-1.1
Albumin	2 g/dL	3.5-5.5 g/dL

Complete blood count

As summarized in Table 2, the complete blood count showed normocytic anemia, with a hemoglobin level of 9 g/dL and a reduced erythrocyte count of 3.8 million/mm³. Leukocyte count was within normal limits at 4,500 cells/mm³, with no significant abnormalities in the differential count. The platelet count was slightly decreased at 130,000/mm³, indicating mild thrombocytopenia, which is commonly seen in cirrhosis due to hypersplenism. Glycemic control was suboptimal, as evidenced by an elevated hemoglobin A1c (HbA1c) of 8%, reflecting poorly managed type 2 diabetes mellitus.

**Table 2:** Complete blood count results indicating normocytic anemia and mild thrombocytopenia. The elevated HbA1c reflects poor glycemic control. These findings are consistent with anemia of chronic disease and hypersplenism, often observed in advanced liver disease WBC: white blood cell; HbA1c: hemoglobin A1c

Test	Observed value	Reference range
Erythrocyte count	3.8 million/mm^3^	4.3-5.9 million/mm^3^
Hemoglobin	9 g/dL	13.5-17.5 g/dL
WBC	4500 cells/mm^3^	4500-11,000 cells/mm^3^
Neutrophils	54%	54-62%
Monocytes	3%	3-7%
Basophils	0%	0-0.75%
Eosinophils	1%	1-3%
Lymphocytes	25%	25-33%
Platelet count	130,000/mm^3^	150,000-400,000/mm^3^
HbA1c	8%	<5.7%

Renal function tests

Renal function tests demonstrated markedly elevated serum creatinine and blood urea nitrogen (BUN) levels, indicative of AKI in the setting of cirrhosis. Urine studies revealed low urinary sodium concentration, low urine osmolality, and a fractional excretion of sodium (FeNa) of less than 1%, which are characteristic of functional renal failure seen in HRS. These findings support the diagnosis of type 1 HRS, which is defined by rapidly progressive renal impairment in the absence of structural kidney disease. Detailed laboratory values are presented in Table 3.

**Table 3:** Laboratory findings characteristic of hepatorenal syndrome. The patient demonstrates elevated serum creatinine and BUN, low urinary sodium, low urine osmolality, and FeNa <1%, indicating a prerenal etiology of acute kidney injury. Additionally, hyponatremia, mild hyperkalemia, and low bicarbonate levels support the presence of hepatic decompensation with secondary renal dysfunction, consistent with hepatorenal syndrome BUN: blood urea nitrogen; FeNa: fractional excretion of sodium

Test	Observed value	Reference range
Serum creatinine	3.8 mg/dL	0.6-1.3 mg/dL
BUN	80 mg/dL	7-20 mg/dL
FeNa	<1%	>1% suggests intrinsic renal etiology (e.g., acute tubular necrosis)
Urinary sodium	8 mEq/L	>20 mEq/L
Urine osmolality	50 mOsm/kg	300-900 mOsm/kg
Serum sodium	122 mEq/L	135-145 mEq/L
Potassium	5.2 mEq/L	3.5-5 mEq/L
Chloride	91 mEq/L	98-107 mEq/L
Bicarbonate	18 mEq/L	22-28 mEq/L

Blood electrolyte panel 

As described in Table 4, the electrolyte panel showed hyponatremia, with a sodium level of 122 mEq/L, and mild hyperkalemia, with potassium at 5.2 mEq/L. Chloride was slightly reduced at 91 mEq/L, and bicarbonate was low at 18 mEq/L, suggesting a mixed electrolyte disturbance. These abnormalities are commonly seen in advanced cirrhosis and HRS and may reflect both impaired renal solute handling and systemic metabolic derangements.

**Table 4:** Electrolyte abnormalities observed in the patient, including hyponatremia and mild hyperkalemia. Chloride was slightly reduced and bicarbonate was low, indicating a mixed electrolyte disturbance. These findings are typical in advanced cirrhosis and hepatorenal syndrome, reflecting impaired renal solute handling and systemic metabolic imbalance

Electrolyte	Observed value	Reference range
Sodium	122 mEq/L	135-145 mEq/L
Potassium	5.2 mEq/L	3.5-5 mEq/L
Chloride	91 mEq/L	98-107 mEq/L
Bicarbonate	18 mEq/L	22-28 mEq/L

Arterial blood gas analysis 

Arterial blood gas analysis revealed a primary metabolic acidosis with partial respiratory compensation, a finding commonly observed in HRS due to impaired renal acid excretion and lactate accumulation from poor tissue perfusion. Despite the presence of acidosis, oxygenation was preserved, and no hypoxemia was noted. Additionally, anemia was observed, which is frequently seen in patients with advanced cirrhosis and renal dysfunction. These findings are summarized in Table 5.

**Table 5:** The patient's arterial blood gas and oxygenation profile, demonstrating mild acidemia with reduced bicarbonate and base excess, indicating a primary metabolic acidosis. A normal PCO₂ suggests partial respiratory compensation. Oxygenation was preserved, although low hemoglobin levels may contribute to borderline tissue oxygen delivery in the context of chronic liver disease and anemia

Parameter	Observed value	Reference range
pH	7.30	7.35-7.45
Bicarbonate	17 mEq/L	22-28 mEq/L
Total CO₂	16 mEq/L	22-29 mEq/L
Standard bicarbonate	18 mEq/L	22-26 mEq/L
Base excess	-5 mEq/L	-2 to +2 mEq/L
PCO₂	35 mmHg	35-45 mmHg
PO₂	80 mmHg	80-100 mmHg
O₂ saturation	94%	>94%
Hemoglobin	9 g/dL	13.5-17.5 g/dL (male)

Ascitic fluid analysis

Diagnostic paracentesis revealed clear ascitic fluid with a serum-ascitic albumin gradient (SAAG) of 1.4 g/dL, consistent with portal hypertension. The low total protein, preserved glucose, white blood cell count below the threshold for spontaneous bacterial peritonitis, and negative cultures ruled out infection and supported a diagnosis of uncomplicated cirrhotic ascites, as described in Table 6.

**Table 6:** Ascitic fluid analysis obtained via diagnostic paracentesis, showing clear fluid, a SAAG of 1.4 g/dL, and low total protein, all consistent with transudative ascites due to portal hypertension in cirrhosis. Glucose was preserved, and the WBC count was below the threshold for SBP, with negative cultures, ruling out infection and supporting the diagnosis of uncomplicated cirrhotic ascites SAAG: serum-ascitic albumin gradient; WBC: white blood cell; SBP: spontaneous bacterial peritonitis

Parameter	Observed value	Interpretation
Appearance	Clear	Non-infected
SAAG	1.4 g/dL	>1.1 → portal hypertension
Total protein	0.8 g/dL	Transudate
Glucose	52 mg/dL	Normal
WBC	200 cells/mm³	<250 → no SBP
Culture and Gram stain	Negative	Negative

Ultrasound of the abdomen

**Figure 1:**
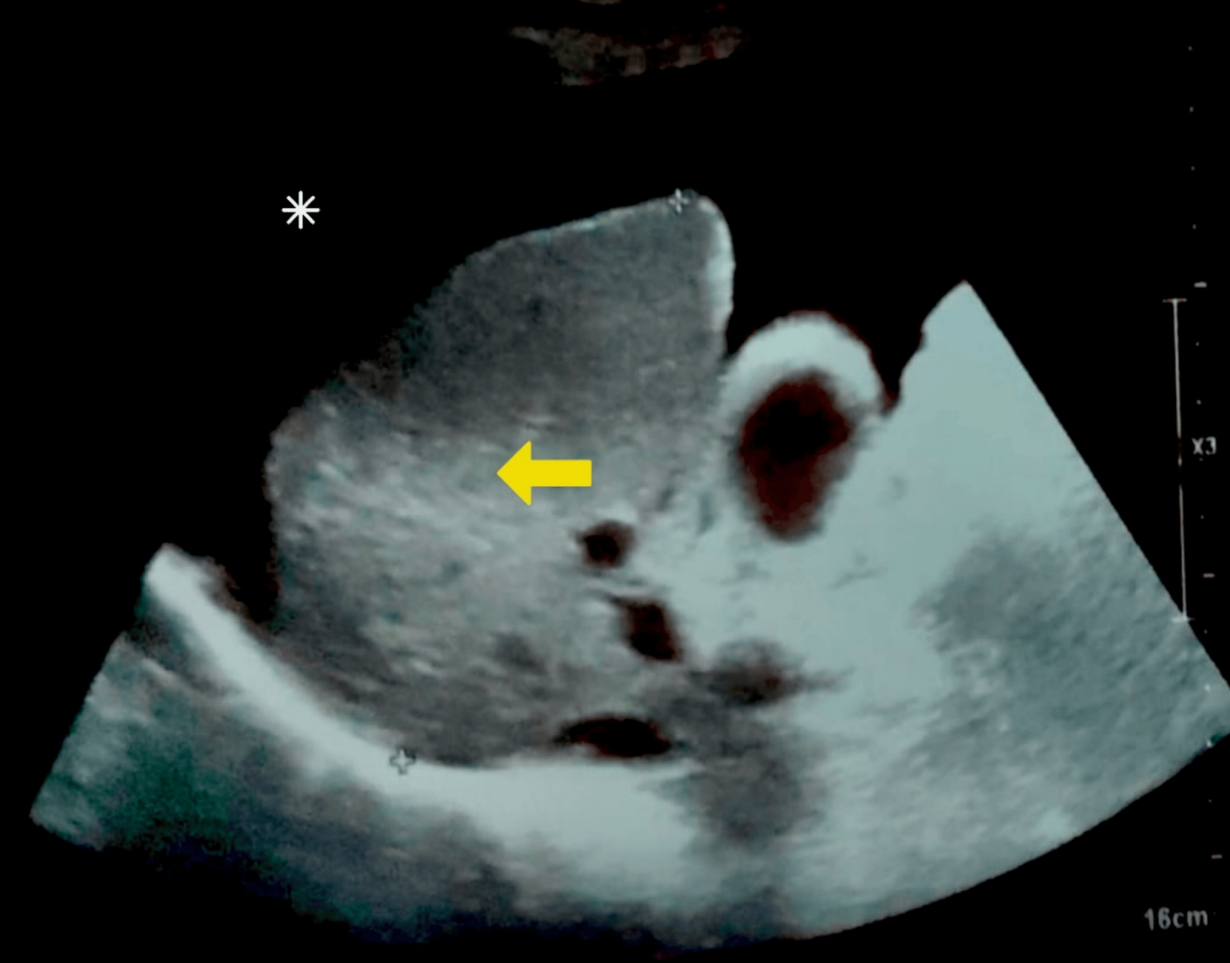
Grayscale ultrasound image of the liver. The yellow arrow indicates the coarse echotexture of the liver, suggestive of underlying cirrhosis. The white asterisk denotes free fluid in the peritoneal cavity, consistent with ascites

Abdominal ultrasonography was performed to evaluate for complications related to cirrhosis. As shown in Figure 1, the liver demonstrated structural changes consistent with chronic liver disease, along with the presence of ascites, suggestive of hepatic decompensation.

**Figure 2:**
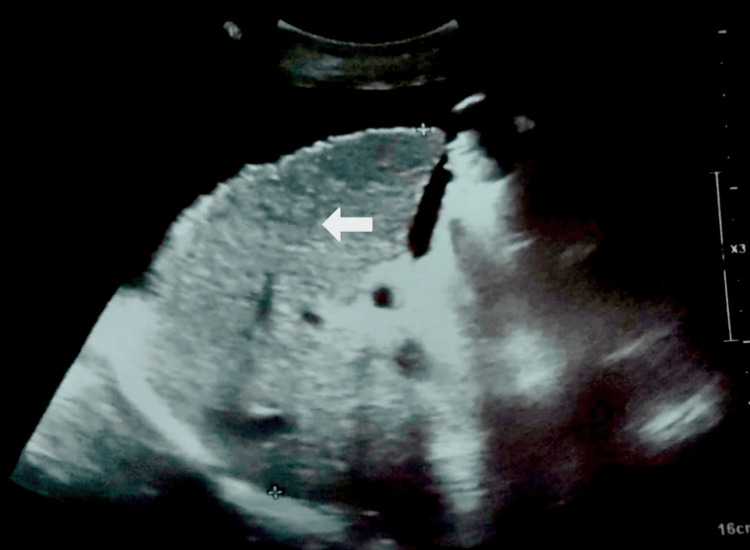
Right upper quadrant grayscale ultrasound image showing multiple hypoechoic nodules within the liver parenchyma on a background of coarse echotexture, findings suggestive of cirrhosis with nodular regenerative changes. The white arrow indicates the hypoechoic nodules

A more detailed view of the liver in Figure 2 revealed imaging features typical of cirrhosis with nodular regenerative changes, further confirming the diagnosis. 

**Figure 3:**
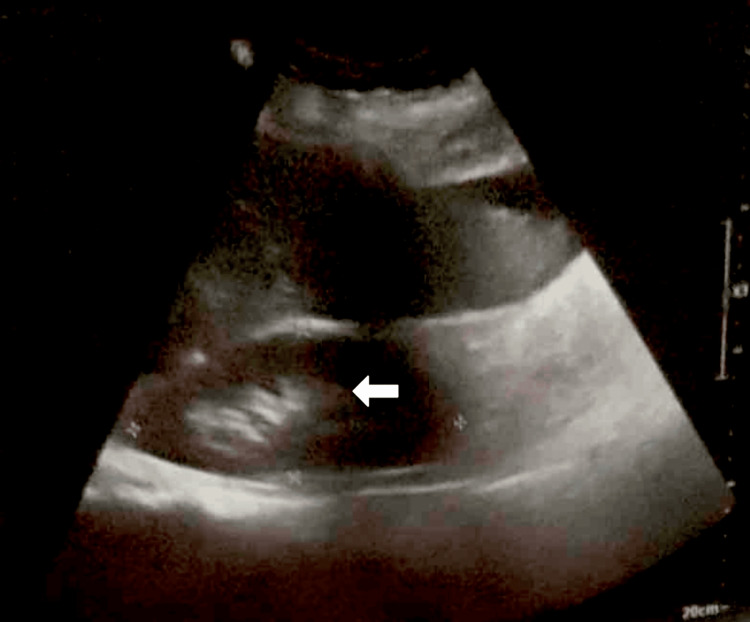
Ultrasound image of the right kidney demonstrating preserved corticomedullary differentiation and normal echotexture, indicating no structural abnormalities. The white arrow highlights the intact corticomedullary interface

To rule out intrinsic renal causes of AKI, a renal ultrasound was obtained. Figure 3 demonstrates a normal right kidney with preserved architecture, supporting the diagnosis of HRS as a functional, rather than structural, renal failure. 

Echocardiogram

**Figure 4:**
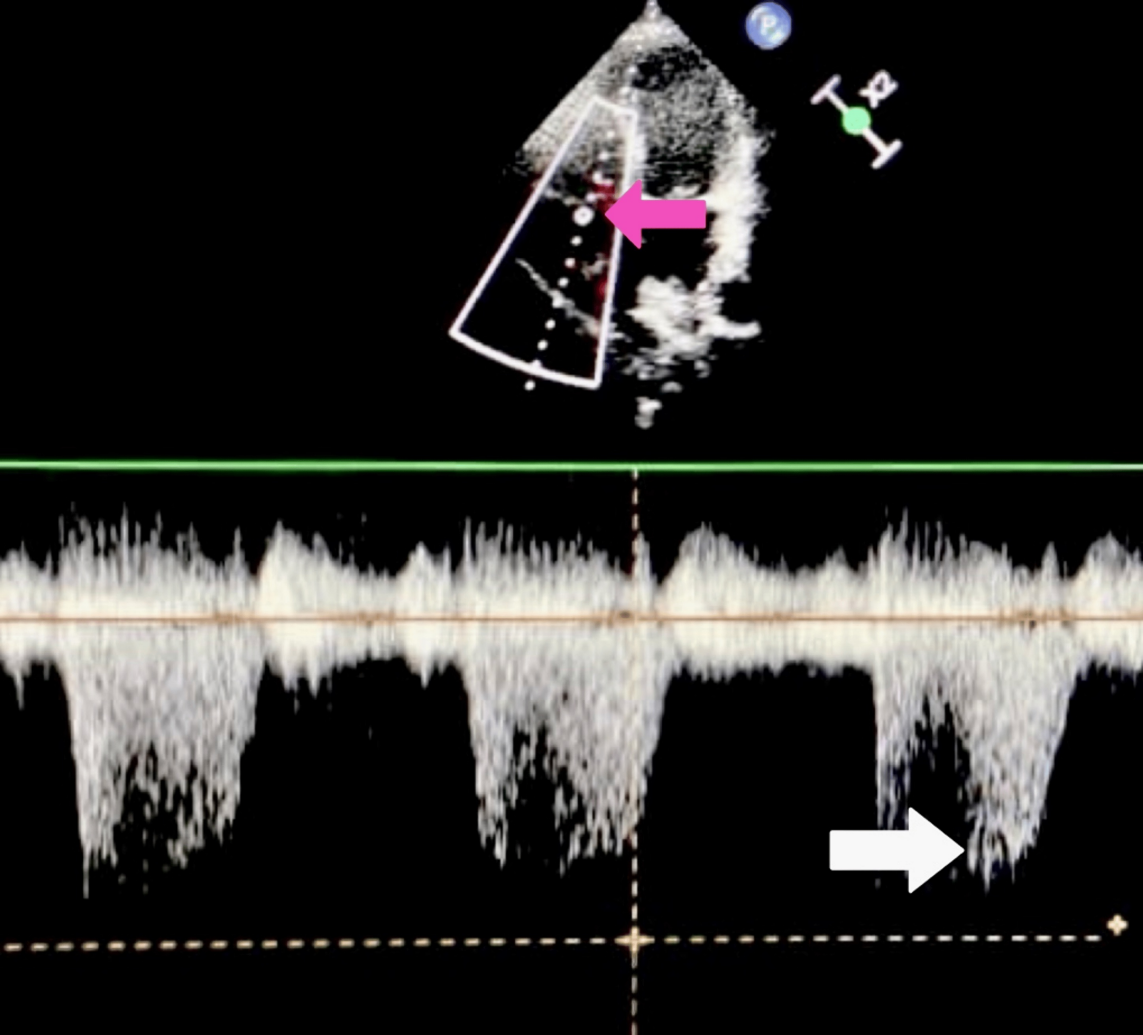
Continuous-wave Doppler tracing of tricuspid regurgitation from the apical four-chamber view. The dense, high-velocity jet envelope (white arrow) represents tricuspid regurgitant flow, indicating elevated right ventricular systolic pressure, suggestive of mild pulmonary hypertension. The pink arrow indicates flow across the tricuspid valve. These findings support the hyperdynamic circulatory state commonly seen in cirrhosis and hepatorenal syndrome

Transthoracic echocardiography was performed to assess for hemodynamic abnormalities associated with advanced liver disease. As shown in Figure 4, the continuous-wave Doppler tracing of tricuspid regurgitation revealed a high-velocity regurgitant jet, indicative of elevated right ventricular systolic pressure and suggestive of mild pulmonary hypertension. This finding supports the presence of a hyperdynamic circulatory state, which is frequently observed in patients with cirrhosis and HRS.

These observations were further supported by dynamic imaging. Video 1 demonstrates moderate tricuspid regurgitation with a prominent high-velocity jet on continuous-wave Doppler, reinforcing the diagnosis of mild pulmonary hypertension and highlighting the hemodynamic consequences of decompensated liver disease.

**Video 1:** Apical four-chamber transthoracic echocardiography demonstrates moderate tricuspid regurgitation with a high-velocity jet on continuous-wave Doppler, indicating elevated right ventricular systolic pressure. These findings suggest mild pulmonary hypertension and a hyperdynamic circulatory state, characteristic of advanced liver disease and consistent with the hemodynamic alterations seen in hepatorenal syndrome

Diagnosis 

The patient is diagnosed with type 1 HRS, complicated by portal hypertension and ascites. The diagnosis is supported by the patient's clinical presentation of reduced urine output, progressive fatigue, and laboratory findings of worsening renal function (elevated serum creatinine and BUN), along with the characteristic features of cirrhosis with ascites seen on ultrasound. The patient's worsening renal function, despite volume resuscitation, and low urine sodium excretion, along with a positive SAAG, further confirm the diagnosis of HRS.

Management and treatment 

This case was managed according to the ICA guidelines, with goals of improving renal perfusion, stabilizing hemodynamics, and bridging the patient to liver transplantation. Diuretics were discontinued to prevent further renal compromise. To differentiate prerenal AKI from HRS, an albumin challenge test (1 g/kg/day for two days, up to 100 g/day total) was administered. Despite fluid resuscitation, the patient's serum creatinine remained elevated at 3.8 mg/dL, supporting the diagnosis of HRS.

Terlipressin was initiated at 0.5 mg intravenously every six hours and titrated up to 2 mg every four hours. This was combined with daily intravenous albumin (20-40 g/day) to enhance oncotic pressure and renal perfusion. After 48 hours, creatinine decreased modestly to 3.4 mg/dL. Given the partial response, therapy was continued with monitoring. The patient was evaluated for ischemic complications through regular assessments of peripheral perfusion and vital signs; no adverse effects were noted. Although norepinephrine was considered as an alternative vasopressor in case of non-response or contraindications to terlipressin, it was not needed. The patient was referred for liver transplantation with a Model for End-Stage Liver Disease (MELD) score of 32; listing was initiated, but waiting time remains pending based on organ availability.

Supportive care included correction of electrolyte imbalances, hyponatremia, hyperkalemia, and metabolic acidosis, while avoiding fluid overload. Given the high risk of spontaneous bacterial peritonitis and sepsis in HRS, prophylactic intravenous ceftriaxone was administered despite negative cultures.

## Discussion

HRS is a life-threatening complication of end-stage cirrhosis, characterized by excessive splanchnic vasodilation, a hyperdynamic circulatory state, and reduced effective blood volume. These changes trigger the activation of vasoconstrictor mechanisms, leading to severe renal vasoconstriction and a marked decline in glomerular filtration rate (GFR) [1].

Pathophysiology

Recent studies emphasize the key role of systemic inflammation in the progression of HRS, particularly in advanced cirrhosis [1]. In early cirrhosis, mild splanchnic vasodilation leads to decreased systemic vascular resistance, which is compensated for by increased cardiac output. However, as cirrhosis advances, elevated levels of vasodilatory mediators, such as nitric oxide and prostaglandins, cause pronounced vasodilation that exceeds cardiac compensatory capacity. This results in effective arterial hypovolemia despite an overall increase in plasma volume [2]. In response, renal and femoral vascular resistance increases, yet renal perfusion remains persistently impaired. This leads to reduced GFR and progressive sodium and water retention, with urinary sodium excretion often falling below 10 mEq/day in advanced stages. Mean arterial pressure may decline significantly, even in the presence of severe renal vasoconstriction. The renin-angiotensin-aldosterone system (RAAS) becomes highly activated, further contributing to circulatory collapse and renal dysfunction [3].

Management 

Pharmacological therapy, which aims to mitigate splanchnic vasodilation and restore effective arterial volume, is the cornerstone of HRS management. When combined with intravenous albumin, terlipressin is the first-line treatment and has been shown to be more effective than other vasoconstrictors at reversing HRS. Close clinical monitoring is necessary due to the significant side effects of this therapy, which include bowel and myocardial ischemia [4,5]. Alternatives like norepinephrine or the combination of midodrine and octreotide can be considered and have demonstrated some success in non-ICU settings or in situations where terlipressin is not available [5].

TIPS can improve renal perfusion and lower portal pressure in certain patients. It is typically only used for patients with preserved liver function who do not have significant encephalopathy or cardiopulmonary contraindications, but it is linked to an increased risk of hepatic encephalopathy [6]. The main purpose of KRT is to provide a supportive transition to liver transplantation, especially for patients who have refractory volume overload, severe electrolyte imbalances, or declining renal function. However, KRT by itself does not increase survival unless it is accompanied by a final course of treatment [7]. At first, liver support technologies like albumin dialysis seemed promising for managing HRS. However, their widespread use was limited when subsequent large-scale studies failed to demonstrate any discernible improvement in survival when compared to conventional KRT [8].

The only proven cure for HRS is still liver transplantation. After a transplant, many patients have substantial renal recovery, especially if their dialysis dependence is low or their renal impairment has not persisted. However, it is still challenging to predict postoperative renal recovery. When evaluating long-term renal outcomes, clinical factors like the length of renal dysfunction, the need for dialysis prior to transplantation, and intraoperative hemodynamic parameters are crucial [9].

Importance of early diagnosis of HRS and markers of AKI recovery 

Early and accurate diagnosis of HRS is critical for improving outcomes in patients with advanced liver disease. Traditional markers such as urine output and urinary sodium excretion are often unreliable in cirrhosis, where renal hypoperfusion and sodium retention can occur even in the absence of structural kidney damage [10]. Recent research has focused on novel biomarkers that may allow earlier and more accurate detection of AKI in cirrhotic patients. Biomarkers such as neutrophil gelatinase-associated lipocalin (NGAL), kidney injury molecule-1 (KIM-1), liver-type fatty acid binding protein (L-FABP), and interleukin-18 (IL-18) have shown potential in distinguishing true HRS from prerenal AKI by reflecting underlying tubular injury. However, their clinical utility remains limited due to undefined cutoff values, low specificity in advanced liver disease, and lack of large-scale validation [11]. Looking ahead of other emerging markers such as cystatin C, tissue inhibitor of metalloproteinases-1 (TIMP-1), and osteopontin, they may play a role in identifying patients at risk of poor renal recovery, particularly after liver transplantation. When integrated with patient-specific factors, these biomarkers may enhance decision-making related to transplant eligibility, timing, and postoperative management [12]. Future research should focus on validating these biomarkers for distinguishing prerenal AKI from true HRS and incorporating them into clinical scoring systems to support timely diagnosis and intervention.

Despite the strengths of this case, there are certain limitations to acknowledge. First, upper gastrointestinal endoscopy was not performed due to the patient's hemodynamic instability and absence of overt gastrointestinal bleeding. Second, although non-selective beta blockers are standard in the management of portal hypertension, they were not part of the patient's treatment regimen, and no documented reason was available for their omission. Lastly, while emerging biomarkers such as NGAL, cystatin C, and KIM-1 may aid in the early diagnosis of HRS, they were not utilized in this case due to institutional resource constraints.

## Conclusions

This case highlights the difficulties in diagnosing and treating patients with alcohol-related decompensated cirrhosis who have HRS. Given the patient's AKI, which is demonstrated by low urinary sodium, high creatinine levels, and positive results from ascitic fluid analysis, it is imperative that HRS be promptly identified using both clinical symptoms and laboratory testing. Some renal improvement was observed after administering terlipressin and albumin, highlighting the significance of early vasoconstrictor therapy. However, the need for liver transplantation remained, indicating that although these pharmaceutical treatments can offer short-term respite, they are not a long-term fix. In order to improve patient outcomes, this case emphasizes the significance of early diagnosis, timely supportive care initiation, and prompt transplant evaluation. Future research should aim to refine diagnostic markers and pinpoint factors that predict renal recovery after transplantation.
